# Efficacy and safety of pegylated interferon in the treatment of JAK2^V617F^-positive polycythemia vera with a dose de-escalation strategy: a single-center retrospective study

**DOI:** 10.3389/fonc.2025.1586839

**Published:** 2025-05-14

**Authors:** Long Chang, Wen-Xin Li, Hao Cai, Jian Li, Ming-Hui Duan

**Affiliations:** ^1^ Department of Hematology, Peking Union Medical College Hospital, Chinese Academy of Medical Sciences & Peking Union Medical College, Beijing, China; ^2^ State Key Laboratory of Complex Severe and Rare Diseases, Peking Union Medical College Hospital, Beijing, China

**Keywords:** polycythemia vera, pegylated interferon, JAK2v617F mutation, dose de-escalation strategy, hematological and molecular response

## Abstract

**Introduction:**

Although pegylated interferon (PEG-IFN) has been widely used in the treatment of polycythemia vera (PV), there is still a significant variability in its specific dosage and administration.

**Methods:**

This single-center retrospective study assessed the efficacy and safety of PEG-IFN in JAK2^V617F^-positive PV patients using a dose de-escalation strategy.

**Results:**

From 2018 to 2022, 110 PV patients received PEG-IFN treatment and monitored for JAK2^V617F^ variant allele frequency (VAF) over 12 months, with 95.4% achieving complete hematological response (CHR) and 70.8% and 71.8% achieving molecular response (MR) according to the ELN2009 and 2013 criteria respectively. Patients with increased Immunoglobulin level after treatment seemed to have a higher MR rate according to the ELN2013 criteria, but the statistical difference was not significant. According to the 2013 criteria, patients with a baseline JAK2^V617F^ VAF ≥75% had a significantly lower MR rate, and those who achieved MR had a significantly lower neutrophil-to-lymphocyte ratio (NLR) after 3 months of treatment. Although 98.2% patients experienced laboratory adverse events, only 6 patients stopping due to adverse reactions.

**Discussion:**

The study found that initiating PEG-IFN at 180ug weekly and adjusting only for adverse events was well-tolerated and may offer superior outcomes to traditional dosing strategies. The 12-month hematological and molecular efficacy were promising, suggesting this approach has the potential to improve long-term survival in PV patients, although further research is needed to confirm these findings.

## Introduction

Polycythemia vera (PV) is one of three classic Philadelphia chromosome-negative myeloproliferative neoplasms, with over 95% of PV patients harboring the JAK2^V617F^ mutation (1). PV is characterized by clonal proliferation of bone marrow hematopoietic stem cells leading to an increase in all blood cells, often accompanied by significant thrombotic risk. Research indicates that controlling erythrocytosis through phlebotomy to reduce hematocrit (HCT) can markedly decrease the risk of thrombotic events, making HCT control a key therapeutic goal for PV, with phlebotomy being a recommended method for managing HCT (2). Due to the limitations of medical insurance policies in our country, phlebotomy is extremely difficult to implement. Moreover, the REVEAL study also found that even with HCT control achieved through phlebotomy, leukocytosis and thrombocytosis remain independent risk factors for increased thrombotic risk (3). Therefore, achieving complete hematological response (CHR) through cytoreductive therapy may be key to further reducing thrombotic risk. Recent studies have also shown that the level of JAK2^V617F^ variant allele frequency (VAF) is a predictive factor for thrombotic risk, suggesting the need to alter treatment strategies for PV patients to aim for molecular response (MR) and thereby minimize thrombotic risk (4–6). Moreover, treatment relying solely on phlebotomy may not be sufficient to reduce the risk of PV patients progressing to bone marrow fibrosis or acute leukemia. SEER data show that the overall survival (OS) of PV patients in the United States, who are primarily treated with phlebotomy, is significantly lower than that of age-matched individuals, highlighting the importance of cytoreductive therapy in PV treatment (7). Hydroxyurea (HU) is currently the most commonly used cytoreductive drug, but it has a low rate of CHR, safety issues with long-term use, such as the risk of secondary malignancies, and almost no sustained molecular response, making it difficult to achieve the goals of delaying fibrosis and prolonging life (8). Research has found that interferon can suppress TNF-α expression in PV patients’ hematopoietic progenitor cells, inhibit the malignant clonal proliferation of PV, and repair the polyclonality of hematopoietic cells, indicating that interferon may be an effective drug for PV treatment (9, 10). Regular interferon is inconvenient to use, requiring injections every other day, and up to 30% of patients have to discontinue treatment due to adverse events(AEs) (11). Pegylated interferon(PEG-IFN) and ropeginterferon have improved these drawbacks, allowing for the use of relatively high doses over the long term while reducing AEs, making them the main choices for cytoreductive therapy in PV (4, 12–14). Recent studies suggest that cytoreductive therapy with interferon not only significantly increases the rate of CHR and further reduces the thrombotic risk in PV patients but also reduces JAK2^V617F^ VAF, achieving a certain level of MR, thereby significantly reducing the risk of disease progression and extending survival (4, 8, 15). Traditionally, interferons are started at low doses and the dose is not increased after achieving HCT or CHR (16). Recent research indicates that the therapeutic effect of interferon may be related to the intensity of the initial dose (4, 15). Therefore, for all PV patients, the strategy of starting with a high dose and only adjusting the dose when AEs occur, can it further improve the CHR and MR efficacy in PV patients’ treatment without increasing AEs, we conducted a preliminary retrospective single center analysis. This study is part of a multicenter clinical trial, which includes both prospective and retrospective data, with the registration number ChiCTR2200065811.

## Methods

### Participants

This retrospective analysis included patients with JAK2^V617F^-positive PV who received treatment with PEG-IFN (Peg-IFN-a-2b; PegBeron, Y shape, 40 kDa, Xiamen Amoytop Biotech, China) at Peking Union Medical College Hospital from January 2018 to December 2022. All patients were diagnosed according to the World Health Organization diagnostic criteria for 2016. Both the inclusion criteria and exclusion criteria can be found on the clinical research website. Before starting PEG-IFN treatment, patients completed baseline assessments including blood cell count and classification, HCT, bone marrow aspiration smear, bone marrow biopsy, next-generation sequencing to determine driver and non-driver gene mutations, and spleen size. An enlarged spleen was defined as palpable below the costal margin on physical examination and B-ultrasound. Written informed consent was obtained from all participating patients. This study was conducted in compliance with the ethical standards of the responsible institution on human subjects as well as with the Helsinki Declaration.

### Treatment

Patients who had never received any drug treatment and voluntarily accepted PEG-IFN treatment were defined as first-line treatment. Patients who had previously undergone phlebotomy, leukapheresis, hydroxyurea, or conventional interferon treatment, and then switched to PEG-IFN treatment due to drug resistance or intolerance, were defined as second-line or subsequent lines of treatment. The definition of drug resistance or intolerance referred to the European LeukemiaNet (ELN) criteria. Patients treated with PEG-IFN alone started with a dose of 180ug once a week. If tolerable, the dose of 180ug once a week was maintained. If combined with ruxolitinib treatment, the starting dose of PEG-IFN was 90ug once a week. All patients received preemptive nonsteroidal anti-inflammatory drugs to prevent adverse reactions before treatment. If grade ≥1 drug-related AEs occurred, the dose and frequency of PEG-IFN treatment could be adjusted according to the situation. If grade ≥3 drug-related AEs occurred, PEG-IFN treatment would be suspended. For AEs related to liver function abnormalities, commonly used clinical hepatoprotective drugs for enzyme reduction treatment were allowed, including drugs such as Polyunsaturated phosphatidylcholine and Bicyclol. All patients were administered low-dose aspirin in the absence of overt contraindications. For patients who need to reduce the number of red blood cells as soon as possible, phlebotomy can be carried out as appropriate in the early stage of treatment.

### Efficacy assessment

Blood routine tests were performed weekly in the early stages of treatment, and liver and kidney function tests were conducted every two weeks. After the hematology parameters stabilized, the frequency was gradually extended up to once every three months. Every three months, additional assessments of blood smear, thyroid function, immunoglobulins, antinuclear antibodies, and other tests were performed. JAK2 gene quantification was assessed every six months using digital droplet PCR(dPCR). The primary endpoints were hematological response and molecular response. Hematological response included HCT response and CHR: an HCT<45% without the need for additional phlebotomy was considered an HCT response; CHR was defined as meeting all three criteria: HCT<45%, white blood cells<10×10^9^/L, and platelets ≤ 400×10^9^/L. Molecular response was defined according to both ELN2009 and ELN2013 criteria, with MR including molecular complete response and partial response (17, 18). Adverse reactions were assessed according to the CTCAE 5.0 version.

### Sequencing technology

The next-generation sequencing platform used was MGISEQ-2000, with FCL-PE150 sequencing reagent kits for sequencing in Pair-end mode, 150bp read length, and an average sequencing depth of 2000x. Single nucleotide mutations (SNVs) and insertions/deletions (Indels) were detected using Sentieon TNscope. DPCR was performed using the ABI QuantStudio™ 3D PCR 20K chip detection, with results interpreted by the hydrolysis signal of mutation-specific probes, and the PCR system was divided into 20,000 micro-wells for detection. The dPCR results were secondarily analyzed using AnalysisSuite™ software. The formula for calculating the mutant allele burden was JAK2^V617F^/JAK2^V617F^+JAK2 wild type. The primer and TaqMan probe sequences were the same as previously reported (19). The forward primer for JAK2^V617F^ was 5’-AAGCTTTCTCACAAGCATTTGGTTT-3’; the reverse primer for JAK2^V617F^ was 5’-AGAAAGGCATTAGAAAGCCTGTAGTT-3’. The wild-type JAK2^V617^ probe was VIC-TCTCCACAGACACATAC-MGB, while the mutant JAK2^V617F^ probe was 6-FAM-TCCACAGAAACATAC-MGB. The experiment used QuantStudio™ 3D Digital PCR 20K Chip v2 and QuantStudio™ 3D Digital PCR Mastermix (Thermo Fisher). DNA extracted from patient peripheral blood was quantified using a Fluorometer Qubit 3.0 (Thermo Fisher), with 100 ng of DNA added to each dPCR reaction system. Data were analyzed using QuantStudio™ 3D AnalysisSuite™ Software. This sequencing method is able to quantitate JAK2*^V617F^* mutants at a prevalence as low as 0.1%, reaching sensitivity recommended for residual disease monitoring (20).

### Statistical methods

SPSS 24.0 software was used for statistical analysis. Remission rates are presented with descriptive statistics. All patients initiating the study treatment were evaluated for response. Univariate analysis was performed to evaluate differences in proportions by the chi-square or Fisher exact tests where appropriate. Non-numeric data were expressed in percentages and rates. Comparisons between groups were made using T tests. P-values<0.05 were considered statistically significant.

## Results

### Patient characteristics

From January 2018 to December 2022, our hospital treated a total of 378 patients with PV, of whom 131 (34.7%) received treatment with PEG-IFN. Among them, 110 patients were regularly followed up within 12 months and monitored for JAK2^V617F^ VAF over 12 months; their baseline characteristics are detailed in **Table 1**. Of all patients treated with PEG-IFN, 55 (50%) were treated as first-line therapy, 53 (48.2%) as second-line therapy, and 2 (1.8%) as third-line or higher therapy. In prior treatments, 45 patients received hydroxyurea therapy, and 23 received regular interferon therapy. Nineteen patients (17.3%) were treated with a combination of PEG-IFN and ruxolitinib, including 5 who started treatment simultaneously due to self-decisions,13 who were treated with PEG-IFN after ruxolitinib because of failing to achieve CHR, and 1 who was treated with PEG-IFN as first-line therapy for 12 months but then added ruxolitinib due to not achieving a MR; Apart from 9 patients who started with an initial dose of 90ug weekly and were all treated with ruxolitinib combination therapy, all other patients began with 180ug weekly. All patients carried the JAK2 ^V617F^ mutation, with a median VAF of 35.04% (range 0.73%-97.2%). Fifteen patients (13.6%) carried non-driver mutations, the most common of which was TET2 mutation in 6 cases, along with DNMT3A (2 cases), and one case each of ASXL1, ROS1, SH2B3, NOTCH1, GNAS, TP53, IDH1, and JAK2L611S.

**Table 1:** Baseline characteristics of the trial patients.

Characteristics	N(%)
No. of patients	110
Male,n(%)	54(49.1)
Age,years,median(range)	51(21-74)
Previous thromboembolic event	30(27.3)
Low risk	64(58.2)
Presence of splenomegaly	25(22.7)
Presence of non-JAK2^V617F^ mutations,n(%)	15(13.6)
Baseline parameters	
Median JAK2^V617F^ VAF,%(range)	35.04(0.73-97.2)
Median hematoglobin, g/L	167(113-236)
Median platelet count,10^9^/L	584(124-2027)
Median leucocyte count,10^9^/L	10.24(4-32.7)
Median LDH	244(120-570)
Parameters after 1 year of treatment	
Median JAK2^V617F^ VAF,%(range)	9.6(0.17-81.5)
Median hematoglobin, g/L	126(93-161)
Median platelet count,10^9^/L	172(46-589)
Median leucocyte count,10^9^/L	3.62(2.04-10.39)
Median LDH	187(133-441)

### Treatment

During the treatment process, no patients developed bone marrow fibrosis or acute leukemia transformation. One patient experienced a new arterial thrombosis, and no patients were found to have developed a second primary tumor or new connective tissue disease. No patients died or were lost to follow-up. Six patients discontinued PEG-IFN therapy permanently due to adverse reactions (rash, hyperthyroidism, fatigue) and 1 patient paused treatment due to pregnancy and resumed treatment after childbirth. Two patients discontinued PEG-IFN treatment at 1 and 4 months, respectively, due to intolerable adverse reactions, while the rest of the patients discontinued treatment after at least 12 months of PEG-IFN therapy. Therefore, a total of 108 patients could be evaluated for molecular response at one year.

### Efficacy

**Figure 1:**
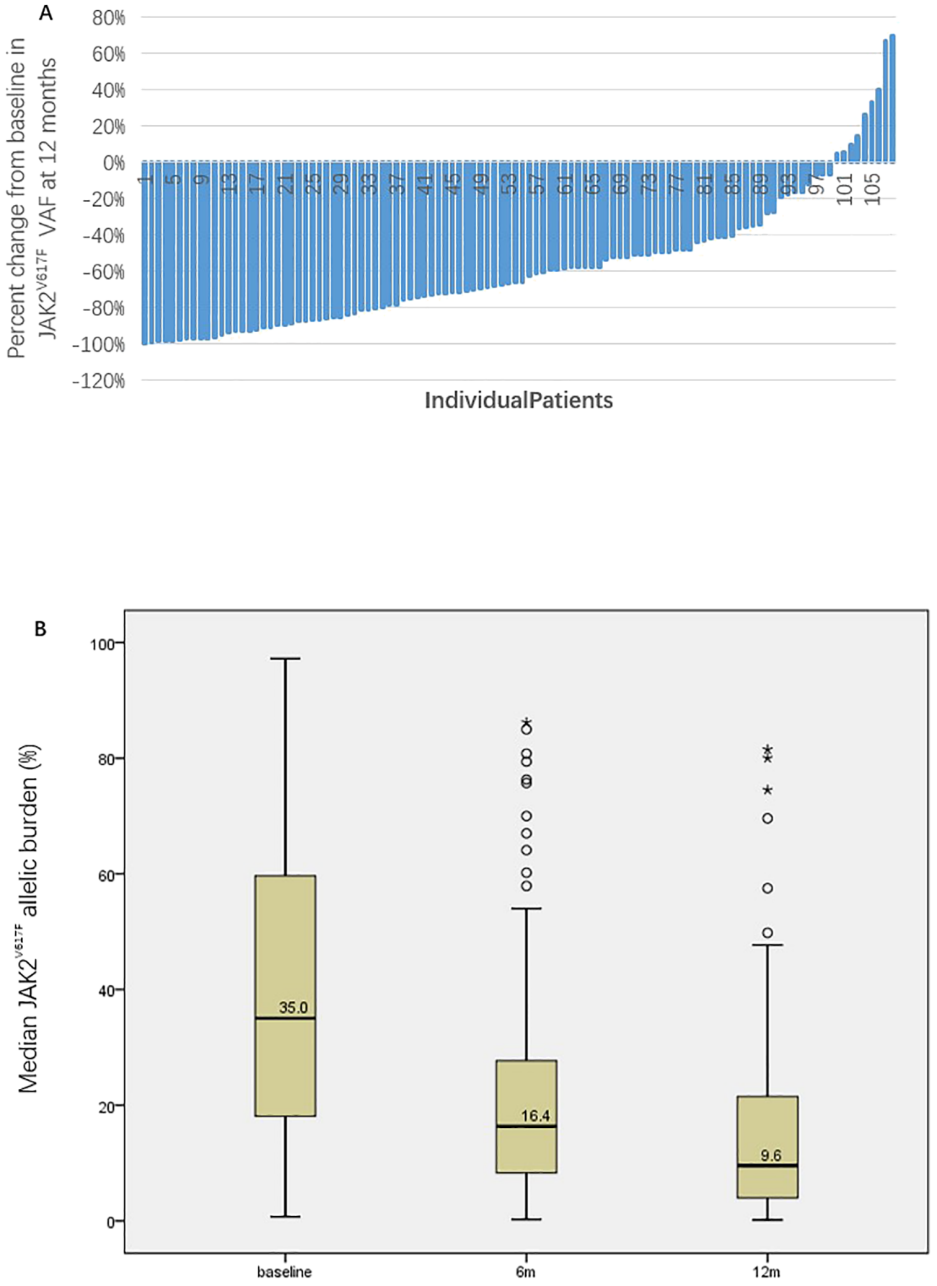
The waterfall plot of the percent change from baseline in JAK2V^617F^ VAF **(A)** and graph demonstrating the change of median JAK2^V617F^ VAF after 12 months of treatment for all patients **(B)**.

Among 108 patients evaluable for efficacy, 103 (95.4%) achieved CHR, 4 (3.7%) achieved HCT response, and 1 (0.9%) did not respond. The waterfall plot of the percent change from baseline in JAK2^V617F^ VAF and graph demonstrating the change of median JAK2^V617F^ VAF after 12 months of treatment for all patients was showed in **Figure 1**. The median JAK2^V617F^ VAF decreased from 35.0% at baseline to 9.6% at 12 months. According to the ELN2009 and 2013 criteria, 96 and 78 patients, respectively, could be evaluated for molecular response, with 6 patients (6.3%) and 4 patients (5.1%) having a JAK2^V617F^ VAF of ≤1%. The number of patients achieving MR were 68 (70.8%) and 56 (71.8%), respectively. Variables such as gender, whether it was first-line treatment, ELN thrombotic risk stratification, presence of splenomegaly at baseline, presence of non-driver mutations, normal LDH levels, and combination treatment with ruxolitinib showed no significant correlation with the MR rate (**Table 2**). The occurrence of hematological adverse events after treatment also showed no significant relationship with MR. Patients with increased Immunoglobulin level after treatment seemed to have a higher MR rate according to the ELN2013 criteria, but the statistical difference was not significant. Baseline JAK2^V617F^ VAF levels and the neutrophil-to-lymphocyte ratio (NLR) were not related to MR. According to the 2009 criteria, patients with a baseline JAK2^V617F^ VAF ≥50% had a significantly lower MR rate. According to the 2013 criteria, patients with a baseline JAK2^V617F^ VAF ≥75% had a significantly lower MR rate, and those who achieved MR had a significantly lower NLR after 3 months of treatment. According to both the 2009 and 2013 criteria, among patients treated with ruxolitinib and PEG-IFN simultaneously, 3 out of 5 (60%) achieved MR, and among those who did not achieve MR after ruxolitinib treatment and then added PEG-IFN, 7 out of 10 (70%) achieved MR, with no significant difference compared to those not treated with ruxolitinib (**Table 2**).

**Table 2:** The 12-month MR according to different ELN criteria and its influencing factors.

Variable	ELN2009 (n=96)			ELN2013 (n=78)		
MR,n (%)	68 (70.8%)			56 (71.8%)		
Non-numeric data,MR,n (%)			P			P
Gender:male vs.female	32 (68.1%) vs. 36 (73.5%)		0.562	25 (71.4%) vs. 31 (72.1%)		0.948
Age:<60vs.≥60	54 (71.1%) vs. 14 (70.0%)		0.927	42 (72.4%) vs.14 (70.0%)		0.836
Treatment line,1^st^ vs. 2^nd^	44 (75.9%) vs. 24 (63.2%)		0.180	39 (76.5%) vs. 17 (63.0%)		0.207
Low vs. high risk	39 (69.9%) vs.29 (72.5%)		0.761	34 (72.3%) vs. 22 (71.0%)		0.895
Splenomegaly:no vs.yes	51 (70.8%) vs. 17 (70.8%)		0.807	40 (69.0%) vs. 16 (80.0%)		0.344
In combination with ruxolitinib: yes vs. no	10 (66.7%) vs. 58 (71.6%)		0.699	10 (66.7%) vs.46 (73.0%)		0.623
Non-driver mutatoin: no vs. yes	56 (67.5%) vs. 12 (92.3%)		0.067	45 (68.2%) vs. 11 (91.7%)		0.096
LDH: normal vs. >ULN	31 (64.6%) vs. 37 (77.1%)		0.178	24 (72.7%) vs. 32 (71.1%)		0.875
Baseline JAK2^V617F^ VAF						
<75%vs.≥75%	61 (72.6%) vs. 7 (58.3%)		0.308	51 (77.3%) vs. 5 (41.7%)		0.012*
<50%vs.≥50%	33 (86.9%) vs. 35 (60.3%)		0.005*	26 (65.0%) vs. 30 (78.9%)		0.171
AE≥grade 1, yes vs. no						
Neutropenia	14 (66.7%) vs. 54 (72.0%)		0.635	39 (69.6%) vs. 17 (77.3%)		0.500
Thrombopenia	48 (66.7%) vs. 20 (83.3%)		0.120	39 (69.6%) vs. 17 (77.3%)		0.500
Anemia	49 (75.4%) vs. 19 (61.3%)		0.155	40 (78.4%) vs. 16 (59.3%)		0.073
Ig increased	28 (75.7%) vs. 40 (67.85)		0.408	27 (81.8%) vs. 29 (64.4%)		0.092
Numeric data,median ± SD						
	MR	NR	P	MR	NR	P
Baseline JAK2^V617F^ VAF	46.43 ± 22.67	36.60 ± 25.99	0.067	49.70 ± 20.15	50.55 ± 26.91	0.894
Baseline NLR	4.44 ± 2.44	4.78 ± 3.98	0.622	4.63 ± 2.55	5.44 ± 4.25	0.306
NLR after 3 months	2.13 ± 1.06	2.66 ± 1.83	0.158	2.10 ± 0.84	3.23 ± 2.17	0.026*

MR, Molecular response; LDH, Lactic dehydrogenase; ULN, Upper limit of normal; AE, Adverse events; Ig, Immunoglobulin; SD, Standard deviation; NLR, Neutrophil-to-lymphocyte ratio; NR, No molecular response.

*p value <0.05.

### Toxicity

Among 108 patients receiving PEG-IFN treatment, 98.2% experienced laboratory adverse events, both hematological and non-hematological (**Table 3**). The most common hematological adverse event was neutropenia. In terms of non-hematological adverse reactions, elevated transaminases were the most frequently reported, with no patients experiencing treatment-related hyperbilirubinemia. Thyroid dysfunction and increased immunoglobulins were also common, but did not lead to discontinuation of PEG-IFN treatment. Other prevalent symptoms included fever (similar to influenza-like symptoms) in 35 cases (31.8%, including 14 cases of grade 3-4), fatigue in 30 cases (27.3%, including 9 cases of grade 3-4), and bone pain in 15 cases (13.6%, including 1 case of grade 3-4). During the treatment, in 35 cases (31.8%), the interval between doses was extended or the dosage was reduced to manage the adverse reactions.

**Table 3:** Adverse events observed in patients who received PEG-IFN.

Event	Grade 1-2	Grade 3-4
Neutropenia, n (%)	77 (70%)	6 (5.4%)
Anemia, n (%)	36 (32.7%)	0
Thrombocytopenia, n (%)	24 (21.9%)	0
Aminotransferase increased, n (%)	87 (79.1%)	1 (0.9%)
Abnormal thyroid function, n (%)	31 (28.2%)	0
Increased immunoglobulin levels., n (%)	44 (40.0%)	0
Fever, n (%)	24 (22.6%)	14 (13.2%)
Fatigue, n (%)	21 (19.8%)	9 (8.5%)
Bone pain, n (%)	14 (13.2%)	1 (0.9%)
Rash, n (%)	9 (8.5%)	1 (0.9%)

## Discussion

According to current guidelines, the treatment goal for PV patients is simply to achieve HCT to reduce the risk of thrombosis (2). However, as a myeloproliferative disorder, recent studies like REVEAL have found that even with HCT control, elevated WBC and PLT counts still independently increase the risk of thrombosis (3). Therefore, achieving CHR may be a more critical treatment goal for reducing the thrombotic risk in PV patients. Further research has found that even with HCT control and CHR, it is still not possible to completely prevent the advanced progression of PV, but it may significantly improve survival prognosis. Data from the SEER database shows that the OS of PV patients in the United States who are treated mainly with phlebotomy and hydroxyurea is significantly lower than that of the normal population of the same age. Real-world data from Weill Cornell Medicine (WCM) on 470 PV patients also shows that PV patients receiving phlebotomy treatment have a significantly lower long-term survival rate, in contrast, the interferon treatment group that can significantly reduce the JAK2^V617F^ VAF has a significantly prolonged OS (8). Therefore, research on further improving the long-term survival of PV patients and on alternative treatment goals that can predict long-term survival is increasing. In the PROUD-PV and MAJIC studies, when using medications such as ropeginterferon or ruxolitinib to treat PV patients, the rate of molecular response was significantly correlated with the rate of CHR at 12 months, and it was also clearly related to progression-free survival (PFS), event-free survival (EFS) (5, 12). It can be seen that if the treatment goal for PV patients is further improved, from HCT to CHR, or even to MR, it may bring better long-term prognosis for PV patients. Therefore, how to effectively and safely reduce the JAK2^V617F^ VAF in PV patients may become one of the important therapeutic goals for PV treatment in the future. Recent studies have shown that IFN treatment for PV can achieve a high rate of CHR and molecular response, some patients’ bone marrow biopsy can significantly improve or even return to normal, a few patients with significantly reduced JAK2^V617F^ VAF can maintain long-term stability of the disease after discontinuing IFN treatment, thus making IFN a significant treatment option for PV patients (3, 21).

To our knowledge, this article represents a summary of the largest case study to date using a dose de-escalation strategy with PEG-IFN for the treatment of PV. Due to a certain incidence of AEs, some patients cannot tolerate IFN treatment. Previous studies have shown that AEs lead to 20-40% of patients being unable to persist with regular IFN therapy (8, 11). The emergence of new IFN formulations such as PEG-IFN and ropeginterferon has significantly changed the tolerance of patients and also made it possible to start treatment with higher doses (4, 15). In the treatment of hepatitis B and C, which commonly present with cytopenia and liver dysfunction, PEG-IFN can generally start at a dose of 180ug weekly, and most patients with viral hepatitis can tolerate it well (22–24). However, in PV where high blood cell counts are the main manifestation, previous studies have started typically at a lower dose and have halted dose escalation after achieving HCT or CHR out of fear of complications such as cytopenia and liver dysfunction, which is actually an irrational approach (8, 12, 16, 25). A series of studies from viral hepatitis have shown that the efficacy of interferon is closely related to dose intensity (24). Therefore, in recent years, the clinical studies of interferon treatment for MPN have gradually increased the dose of interferon. In the PROUD study, although ropeginterferon started at 100ug, the discontinuation rate due to adverse events has been reduced to about 13%. In Jie Jin et al.’s study, ropeginterferon was started directly at 250ug, quickly titrating to the target dose, with no patients discontinuing due to AEs (15). The median time to CHR was only 5.6 months, and the rates of hematologic and molecular response at 52 weeks were significantly higher than those in previous studies using the lower dose of ropeginterferon(**Table 4**). This indicates that starting new IFN treatment at higher doses helps to significantly improve hematologic and molecular efficacy without increasing the incidence of AEs. Our study further increased the intensity of initial treatment, with the vast majority of patients starting at the maximum tolerated dose recommended for PEG-IFN, and a few patients combined with ruxolitinib starting at 90ug weekly, which is also higher than the currently recommended dosage for combination therapy (6, 26). Moreover, not only was the starting dose increased, but during treatment, PEG-IFN was not discontinued if patients did not experience grade 3–4 toxicities, allowing patients to maintain a higher dose intensity throughout the treatment. Through close monitoring and dose adjustment, although there was a higher incidence of AEs, most were grade 1-2, with a low rate of grade 3–4 toxicities, and the rate of discontinuation due to AEs was less than 10%, confirming that the dose de-escalation strategy starting with the highest recommended dose of PEG-IFN is safe for PV patients. Therefore, it can be seen that starting with higher doses of PEG-IFN or ropeginterferon and then reducing the dose when AEs occur is a safe treatment strategy for PV patients, which may help PV patients achieve better hematologic and molecular efficacy.

**Table 4:** In the treatment of PV patients with PEG-IFN or ropeginterferon, the CHR and MR status, as well as the incidence of major hematological adverse events in different studies, are calculated according to the ELN2009 and 2013 criteria, respectively.

Trials	Drug	n	12m CHR(%)	12m MR(%)		Adverse events(%)	
				ELN2009	ELN2013	Neutropenia	Thrombocytopenia
This study	PEG-IFN	108	95.4	70.8	71.8	75.4	21.9
PVN1 (16)	PEG-IFN	37	94.6	58.6	NA	NA	NA
MDACC (25)	PEG-IFN	40	79	61(60m)	NA	NA	NA
NCT 01259856 (27)	PEG-IFN	82	28	NA	NA	20	22
Low PV (13)	ropeginterferon	64	NA	NA	22	NA	NA
PROUD (12)	Ropeginterferon	257	71	34	NA	4	23
CTR20211664 (4)	Ropeginterferon	49	71.4	67.1	43.2	30.6	16.3
COMBI 2 (6)	PEG-IFN+RUXO	25	88	NA	59	60	36

CHR, Complete hematologic response; MR, Molecular response; RUXO, ruxolitinib; NA, not available.

We conducted a preliminary analysis of the predictive factors for MR of PEG-IFN dose de-escalation strategy in PV patients. Factors such as age, ELN thrombosis risk stratification, presence of splenomegaly at baseline, elevated LDH, and presence of HMR were all found to be unrelated to the rate of MR. The PROUD study showed that the MR rate was significantly lower in high-risk compared to low-risk patients, but our results did not show this difference. It is hypothesized that the dose de-escalation strategy may partially overcome the negative impact of high risk on MR. Additionally, previous studies have shown that the presence of non-driver gene mutations leads to poor molecular therapeutic efficacy and worse prognosis. In our study, although only 13.6% of PV patients had various non-driver genes at baseline, the presence of non-driver genes did not significantly affect hematological and molecular therapeutic efficacy. It remains to be further investigated whether this result is related to our dose de-escalation strategy. The DALIAH study showed that interferon therapy, while reducing MPN driver genes, may also have a role in preventing the emergence of non-driver genes, indicating that interferon has an inhibitory effect on non-driver genes (28). Previous studies have reported that PV patients have higher NLR levels than normal individuals, and baseline NLR levels may be associated with long-term prognosis such as thrombotic risk (29). In our study, there was no significant impact of baseline NLR on MR, but patients who achieved MR according to the 2013 criteria had significantly lower NLR levels three months after treatment. NLR may be related to the intensity of interferon therapy, once again suggesting that the dose de-escalation strategy may lead to a higher MR rate. Moreover, our study indicates that, a baseline JAK2^V617F^ VAF of ≥50% may be associated with MR at 12 months according to the ELN2009 criteria; a baseline JAK2^V617F^ VAF of ≥75% may be associated with MR at 12 months according to the ELN2013 criteria, suggesting that a higher baseline JAK2^V617F^ VAF value may make it more difficult to achieve MR.

Some PV patients treated with ruxolitinib can also achieve MR, and which is correlated with prolonged survival (5, 30). Therefore, the MR effect of combining ruxolitinib with PEG-IFN in the treatment of PV is worth further investigation. Some individual studies have shown that this combination therapy may achieve a better MR than monotherapy (6, 31). In our study, a few patients were treated with a combination of ruxolitinib and PEG-IFN in various ways. This included initiating the combination therapy from the start, as well as adding PEG-IFN to patients who had been on long-term ruxolitinib treatment but without unsatisfactory MR. Even though the dosage of PEG-IFN in our combination treatment regime is higher than which reported in previous literatures, combination therapies have not demonstrated a higher MR rate in our study. This may be related to the small sample size of our study, or it could be due to the difficulty of further increasing the MR rate on top of high-dose PEG-IFN treatment. Therefore, whether this combined treatment strategy offers an advantage in MR over high-dose PEG-IFN alone requires further research.

Our study has some limitations. Firstly, being a retrospective study, there is a certain degree of bias in data collection. Additionally, the study includes both first- and second-line treatments for PV patients, as well as a small number of patients treated with the combination of ruxolitinib, which prevents a uniform baseline condition. However, our findings indicate that there is no statistical difference in the rates of CHR and MR between first- and second-line treatments, and the use of ruxolitinib does not seem to affect the MR rate at this time, to some extent compensating for the impact of this bias. Thirdly, this study is a single-center, single-arm cohort study; although the uniformity of diagnosis and treatment helps reduce research bias, future multi-center controlled studies are needed to verify the findings for more reliable conclusions. Fourthly, this study only observes the efficacy and safety data at one year; the rate of grade 1–2 adverse events with this dose de-escalation strategy is relatively high, and its long-term safety remains to be observed. Further follow-up over a longer period is required to ultimately clarify its impact on long-term survival outcomes.

In summary, PEG-IFN treatment directly at 180ug/w and adjusting the dose only when adverse events occur, this dose de-escalation strategy for treating Chinese PV patients is well-tolerated. Its 12-month hematological and molecular efficacy may be superior to traditional dose strategies, and the impact of these short-term surrogate markers on the long-term survival prognosis of PV patients warrants further research.

## Data Availability

The original contributions presented in the study are included in the article, further inquiries can be directed to the corresponding author.
