# Correction: Efficacy and safety of pegylated interferon in the treatment of JAK2^V617F^-positive polycythemia vera with a dose de-escalation strategy: a single-center retrospective study

**DOI:** 10.3389/fonc.2025.1641150

**Published:** 2025-06-19

**Authors:** Long Chang, Wen-Xin Li, Hao Cai, Jian Li, Ming-Hui Duan

**Affiliations:** ^1^ Department of Hematology, Peking Union Medical College Hospital, Chinese Academy of Medical Sciences & Peking Union Medical College, Beijing, China; ^2^ State Key Laboratory of Complex Severe and Rare Diseases, Peking Union Medical College Hospital, Beijing, China

**Keywords:** polycythemia vera, pegylated interferon, JAK2v617F mutation, dose de-escalation strategy, hematological and molecular response

In the published article, there was an error in the **Funding** statement. “The funder National High Level Hospital Clinical Research Funding 2022-PUMCH-B-047 to Ming-hui Duan” was erroneously omitted. The correct **Funding** statement appears below.

“This study was supported by the National High Level Hospital Clinical Research Funding 2022-PUMCH-B-047 to Ming-hui Duan. This study also received funding from Xiamen Amoytop Biotech Co., Ltd. The funder was not involved in the study design, collection, analysis, interpretation of data, the writing of this article or the decision to submit it for publication.”

The authors apologize for this error and state that this does not change the scientific conclusions of the article in any way. The original article has been updated.

